# Dynamic Organization of SecA and SecY Secretion Complexes in the *B*. *subtilis* Membrane

**DOI:** 10.1371/journal.pone.0157899

**Published:** 2016-06-23

**Authors:** Alex Dajkovic, Elizabeth Hinde, Calum MacKichan, Rut Carballido-Lopez

**Affiliations:** 1 Micalis Institute, INRA, AgroParisTech, Université Paris-Saclay, 78350 Jouy-en-Josas, France; 2 School of Medical Sciences, University of New South Wales, Sydney, Australia 2052; J. Heyrovsky Institute of Physical Chemistry, CZECH REPUBLIC

## Abstract

In prokaryotes, about one third of cellular proteins are translocated across the plasma membrane or inserted into it by concerted action of the cytoplasmic ATPase SecA and the universally conserved SecYEG heterotrimeric polypeptide-translocating pore. Secretion complexes have been reported to localize in specific subcellular sites in *Bacillus subtilis*. In this work, we used a combination of total internal reflection microscopy, scanning fluorescence correlation spectroscopy, and pair correlation function to study the localization and dynamics of SecA and SecY in growing *Bacillus subtilis* cells. Both SecA and SecY localized in transient and dynamic foci in the cytoplasmic membrane, which displayed no higher-level organization in helices. Foci of SecA and SecY were in constant flux with freely diffusing SecA and SecY molecules. Scanning FCS confirmed the existence of populations of cellular SecA and SecY molecules with a wide range of diffusion coefficients. Diffusion of SecY as an uncomplexed molecular species was short-lived and only local while SecY complexed with its protein partners traversed distances of over half a micrometer in the cell.

## Introduction

The evolutionarily highly conserved general secretory (Sec) pathway catalyzes the translocation of proteins across or into the cytoplasmic membrane in prokaryotes and the endoplasmic reticulum and thylakoid membranes in eukaryotes (for a recent review see [1]). The protein translocating pore—the translocon—is a heterotrimeric integral membrane complex composed of one large subunit and two small subunits, termed SecY and SecE/G respectively, in prokaryotes [2,3,4,5,6,7,8].

In bacteria, the SecYEG complex associates with different ligands to catalyze client protein translocation. Nascent integral membrane proteins are delivered by the signal recognition particle (SRP) to the SecYEG channel for co-translational insertion. Proteins destined to be secreted across the membrane are post-translationally translocated and assume their stable tertiary structure after translocation. These polypeptide substrates bind chaperones in the cytoplasm, which prevent their folding and/or aggregation, before they are delivered to the ATPase motor protein SecA. SecA is an essential peripheral membrane protein that interacts with the SecYEG pore and helps to catalyze translocation [2,3,4,5,6,7]. The low affinity binding of SecA to acidic phospholipids [9] facilitates its high affinity binding to the SecYEG complex, which together constitute the translocase. Anionic phospholipids, particularly cardiolipin, also stimulate the self-association of SecY and the formation of a high-affinity binding surface for SecA [10,11,12].

When SecA binds to SecYEG, the high-affinity binding of SecA to the pre-protein becomes activated and the SecA-powered translocation commences [12,13]. Recent *in vitro* work has shown that SecA moves polypeptides through the SecYEG channel by a “push and slide” mechanism, i.e. by a combination of passive sliding and ATP-driven pushing, in a moderately processive manner [11]. While some molecules of SecA remain bound to the translocon during a secretion event, there is also a flux of SecA molecules in continuous dissociation and rebinding on the timescale of seconds. This association-dissociation requires the interaction of the N-terminal of SecA with phospholipids and is stabilized by the presence of substrate [11]. However, whether similar accumulation and flux of SecA at the translocon occurs *in vivo* is not known.

The structural and functional properties of the Sec pathway have been studied in great detail *in vitro*, traditionally using bacterial proteins. However, major questions remain unresolved and only recently has the sub-cellular localization of Sec components been visualized in bacteria. Using GFP fusions and immunolabelling visualized by conventional epifluorescence microscopy, SecA and SecY were reported to localize in multiple clusters organized in a helical-like arrangement on the cytoplasmic membrane in the rod-shaped model Gram-positive bacterium *Bacillus subtilis* [14]. SecG and SecE were also reported to localize in helices in *Escherichia coli* [15], although SecE and SecY had been previously reported to be uniformly distributed on the membrane of this organism [16]. In the ellipsoidal (ovococcus) *Streptococcus pyogenes*, SecA and several substrates for the Sec pathway were visualized by immunogold electron microscopy and reported to localize in a single cluster near the division septum, named the “ExPortal” [17,18]. However, a subsequent study on the same organism obtained contrasting results by using both immunogold labelling and immunofluorescence microscopy and showing that two different signal sequences direct secretion of proteins to two different regions of the cell [19]. More recently, SecA and SecY were reported to localize in different parts of the mid-cell region in *Streptococcus pneumoniae* as a function of the cell cycle [20], arguing against the existence of an ExPortal in this organism too.

Altogether, these studies suggested that the the localization of the Sec machinery, unlike the sequence of its protein components, is not conserved. Many of them were performed by immunolabelling on fixed cells and none addressed the dynamics of secretion proteins in living cells. Ideally, non-intrusive labeling methods and techniques allowing real-time visualization of highly dynamic processes are necessary for understanding the dynamic process of translocation of polypeptide chains across the bacterial membrane *in vivo*.

Total internal reflection microscopy (TIRFM) is a sensitive method that provides high temporal resolution of membrane-associated fluorescent signals since the amplitude of the exciting evanescent wave decays with the exponent of 6 into the cytoplasm. It was recently applied to the study of cortical processes in bacterial cells [21,22]. Complementary to TIRFM, methods based on fluorescence correlation spectroscopy (FCS) also give information on the dynamics of proteins *in vivo*. FCS-based methods have single molecule sensitivity and high dynamic resolution, providing information about the diffusion times of fluorescent molecules, their concentration, their binding to various structures, and the diffusive paths they may traverse in the cell. In particular, pair correlation methods have been recently used to characterize and quantify the diffusive paths and barriers to diffusion of fluorescent molecules in eukaryotic cells [23], but have never been applied to bacteria.

In this study we used an unprecedented combination of TIRFM, scanning FCS [24], and pair correlation function (pCF) [23,25] approaches to image the sub-cellular localization and dynamics of the essential components of the secretion machinery SecA and SecY in growing *B*. *subtilis* cells.

## Results

### SecA forms dynamic foci in the membrane

We used TIRFM to visualize the localization of a functional SecA-GFP fusion expressed from the endogenous *secA* promoter, as the only copy of SecA in the cell, in exponentially growing cells of *B*. *subtilis*. The strain carrying this construct has no defects in secretion of alpha amylase and proteases and its doubling time is indistinguishable from the wild-type [14]. To examine the localization and dynamics of SecA-GFP, we acquired time series in streaming mode (integration time of 100 ms, over 1 to 2 min, see S1 Video). To study the cellular organization of SecA-GFP, in addition to snapshot images of 100 ms, we generated maximum projections over characteristic timescales to determine the average structures formed by SecA-GFP in the cell.

**Fig 1:**
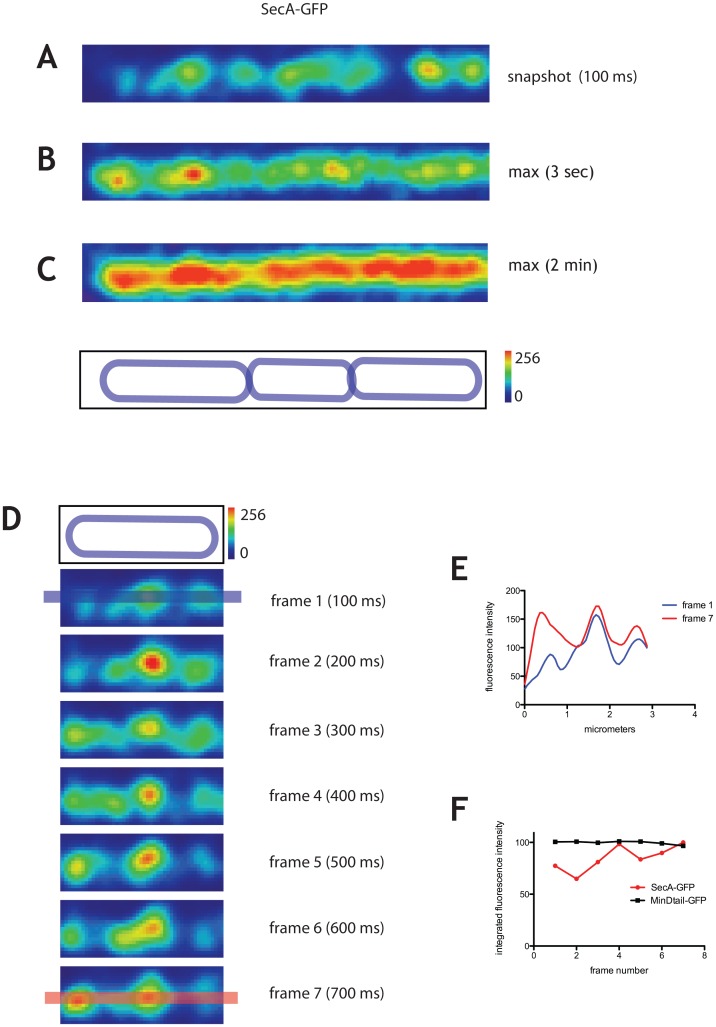
Dynamics of SecA-GFP imaged by TIRF. SecA-GFP (strain secA::pSAG2) imaged by TIRFM. Time series were taken over 2 min in streaming mode using 100 ms integration time. A typical cell is displayed with ‘physics’ LUT from FIJI (see Materials and Methods). **A.** 100 ms snapshot. **B.** and **C.** Typical 3 s **(B)** and 2 min **(C)** maximum projections showing average localization of SecA-GFP over these timescales. The outline of the cells is shown above the panels (see Materials and Methods). The intensity scale for fluorescence is shown on the right of the cell outline panel; it also serves as a scale bar of 1 μm. **D.** Successive frames of the first cell on the left from panel A over a 700 ms time window. The top panel is the cell outline. Intensity scale for fluorescence is 1 μm. **E.** Fluorescence intensity profiles along the line drawn over the long axis of the cell shown for frames 1 and 7, as shown in blue and red respectively in panel D. **F.** Integrated fluorescence intensities for the entire surface of representative cells expressing SecA-GFP (in red) and GFP stably attached to the membrane via the membrane binding tail of MinD (MinDtail-GFP, in black) (see also S1 and S3 Figs).

In individual snapshot images (integration time 100 ms), SecA-GFP localized in distinct foci throughout the membrane (Fig 1A). This is consistent with the previously reported localization of inducible SecA-GFP to bright clusters throughout the membrane except that no helical patterns could be discerned here [14,26]. The average linear density of SecA-GFP foci was 1.8±0.3 (n = 100) focus per micrometer of cell length, indicating an average distance between foci of 0.55 μm. In partial maximum projections of 3 s (Fig 1B), which correspond approximately to the timescale of translocation of one polypeptide across the membrane in reconstituted *in vitro* systems [27], SecA-GFP was found in hotspots more diffuse than the foci observed in the 100 ms snapshots (Fig 1B). Nevertheless, their linear density was identical, 1.8±0.2 (n = 100) clusters per micron of cell length. When the translocation ATPase function of SecA was inhibited by sodium azide [28] 3 s maximum projections did not produce discrete localizations of SecA-GFP, as in untreated cells, but rather a disperse signal over the whole cell (S1 Fig), indicating that the maximum projections are reporting a physiologically relevant localization. Additionally, membrane-attached GFP did not organize in foci at any timescale (see below).

In 3 s, a diffusing protein associated with the membrane would be expected to have moved an average length of ~0.5 to 1.5 μm, given l∼(4Dt)^1/2^, where D = 0.02 to 0.2 μm^2^/s for measured diffusion coefficients for freely diffusing bacterial membrane proteins [29]. This would result in the homogenization of the fluorescence signal in the 3 s maximum projection image and the disappearance of discrete foci. SecA is a peripheral membrane protein which, ceteris paribus, would be expected to diffuse faster than the proteins inserted in the membrane whose diffusion coefficients are cited above. Nevertheless, SecA-GFP foci were easily discerned in 3 s partial maximum projections, indicating that they represent molecular complexes too large to diffuse away over this time scale, or complexes bound to an immobile structure such as the peptidoglycan. The more diffuse localization of SecA-GFP in 3 s maximum projections suggested repeated interaction of SecA molecules with a large structure. SecA hotspots were, however, not always found in the same positions in successive 3 s maximum projections and their apparent lifetimes varied from 3 to 12 s (S2 Fig), indicating that they are dynamic on the membrane over longer time scales and suggesting that they are not attached to peptidoglycan. Consistent with this, in maximum projections over the timescale of 2 min, SecA-GFP fluorescence signal became distributed over the entire membrane surface (Fig 1C), suggesting that there are no preferred cellular addresses for the localization of SecA in the cell.

### Cytosolic SecA molecules dynamically interact with membrane-associated SecA foci

The fluorescence intensity of SecA-GFP foci fluctuated over consecutive 100 ms frames (Fig 1D–1F), which corresponds to the timescale of diffusion and molecular interaction. This fluctuation of fluorescence intensity could be due to: (1) rapid redistribution of membrane-associated SecA-GFP molecules associating/dissociating from the foci, (2) association-dissociation of cytoplasmic molecules of SecA-GFP entering and exiting the TIRF field, or (3) both.

**Fig 2:**
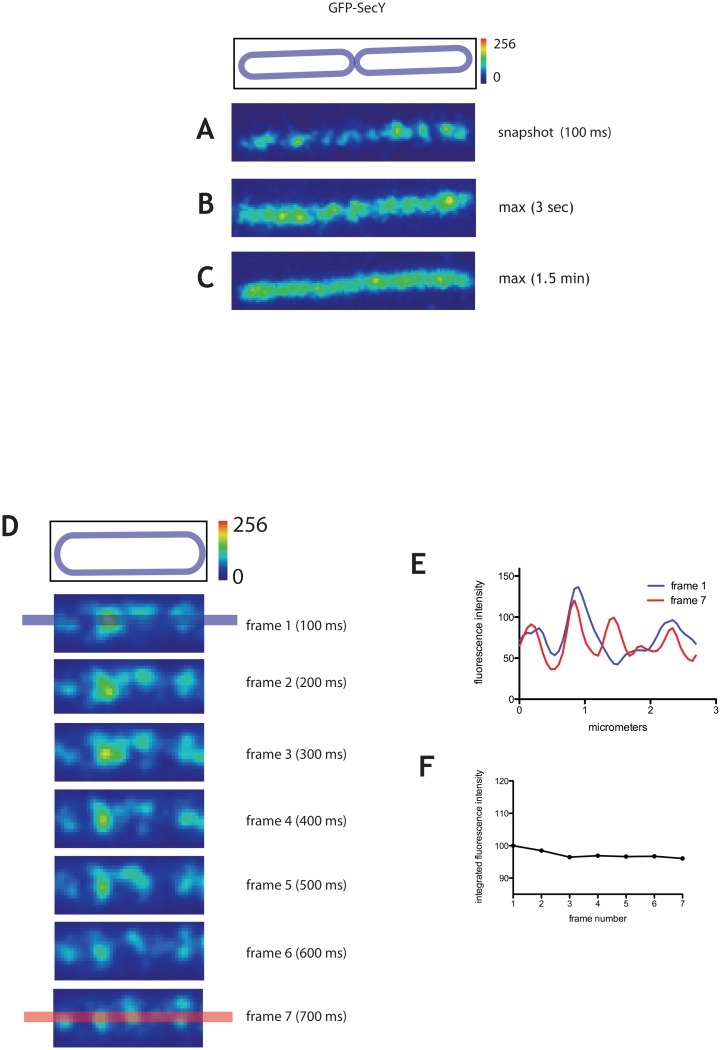
Dynamics of GFP-SecY on the membrane. Distribution of GFP-SecY on the membrane of exponentially growing *B*. *subtilis* cells. Strain amyE::pGY1 (*amyE pxyl-gfp-secY spc*) was grown in LB in the presence of 0.05% xylose. Time series were taken over 1 min in streaming mode using 100 ms integration time. A typical cell is displayed with ‘physics’ LUT from FIJI (see Materials and Methods). **A.** 100 ms exposure TIRF image, **B.** and **C.** maximum projection of a 3 s (**B**) time window and **(C)** 1.5 min time window. The cell outlines are shown above the panels (see Materials and Methods). **D.** Cell outline and successive frames from a time-lapse TIRF acquisition taken in streaming mode. Intensity scale for fluorescence, 1 μm. **E.** Fluorescence intensity profiles along the line drawn over the long axis of the cell for frames 1 and 7, as shown in blue and red respectively, panel G. **F.** Integrated fluorescence intensities for the entire surface of the cell for the frames shown in panel D.

Integrated fluorescence intensity of SecA-GFP on the entire cell surface visible in TIRF section fluctuated appreciably between consecutive 100 ms frames (Fig 1F). Such fluctuations were not detected in cells expressing GFP fused to the membrane-binding tail of *B*. *subtilis* MinD (MinD_tail_), which is composed of an amphipathic helix sufficient to stably attach proteins to the membrane [30] (Fig 1F, see also S1 and S3 Figs). Furthermore, they were also not detected in cells expressing the integral membrane protein SecY fused to GFP (Fig 2F) thus suggesting that changes in axial position of fluorescent proteins in the TIRF field are not the sole origin of fluctuations. For further evidence supporting the idea that cytoplasmic molecules of SecA dynamically interact with clusters in the membrane see Table B in S1 File and Supporting Information. In addition to the dynamic exchange between the membrane and cytoplasmic pools of SecA, there was also a net accumulation of SecA-GFP fluorescence in the foci over several seconds, followed by dissipation (S4 Fig).

Taken together, our results suggest that SecA forms dynamic foci in the membrane that are composed of, or associated with, large molecular complexes with which free SecA molecules dynamically interact.

### SecY dynamically localizes in membrane foci

To gain further insight into the dynamics of the translocon, we next analyzed the dynamics of a GFP-SecY fusion. SecY is the largest component of the translocon, and essential for viability and translocation. Unfortunately, attempts to replace the *secY* gene by *gfp-secY* at the native locus were unsuccessful. Thus, we used the merodiplid *gfp-secY* strain used in previous studies, which contains both the wild-type, unlabeled copy of the *secY* under control of its endogenous promoter at the native locus and a xylose-inducible copy of *gfp-secY* integrated at the ectopic *amyE* locus [14]. However, while high concentrations of inducer (1% xylose) were used in the previous report, here we expressed *gfp-secY* from the lowest xylose concentration allowing GFP-SecY detection in our system (0.05% xylose) to avoid overproduction artifacts. The fluorescence intensity under these conditions was significantly lower than for SecA-GFP (Fig 2A), but sufficient for detection with our microscope set-up. The GFP-SecY fluorescent fusion protein was associated with the membrane, as evidenced by its cortical localization in cell midsections imaged by epifluorescence microscopy (S5A and S5B Fig). Furthermore, it is not toxic when highly overexpressed, suggesting that it may be partially functional (data not shown and [14].

As for SecA-GFP, we acquired timelapse movies with 100 ms integration times (S2 Video) and produced maximum projections over characteristic times to examine the average structures produced by GFP-SecY. In 100 ms snapshots, GFP-SecY displayed a punctate pattern on the membrane similar to SecA-GFP (Fig 2A), and consistent with the localization previously reported, with the exception that we detected no helical patterns [14,31]. Like SecA foci, SecY foci were more diffuse in 3 s maximum projections (Fig 2B), and their lifetimes varied form 3 to 18s, indicating that their localization was not fixed over time (S5C and S5D Fig). Strikingly, the average linear density of GFP-SecY foci (1.8±0.5 per micron of cell length, n = 100) was the same as the spacing measured for the SecA foci. Maximum projections of longer time series indicated that SecY foci were dynamic with no preferred localization in the membrane (Fig 2C).

Like SecA-GFP foci, many GFP-SecY structures reorganized over several hundred milliseconds (700 ms) and changed intensity (Fig 2D and 2E), suggesting that the foci represent molecular assemblies with which the membrane subpopulation of SecY dynamically associates. In support of this, and in contrast to SecA, the integrated fluorescence intensity of GFP-SecY (Fig 2F) in the cell membrane remained constant except for slight photobleaching, as would be expected for membrane-associated proteins (see also Supporting Information and Table B in S1 File). Taken together, these results indicate that GFP-SecY is found in randomly dispersed foci that dynamically assemble and disassemble in the membrane.

### Scanning FCS confirms distinct populations of SecA-GFP and GFP-SecY

To probe the local diffusion coefficients of subpopulations of SecA-GFP and GFP-SecY in the cell, we acquired scanning FCS measurements along the long axis of cells (see Materials and Methods) and carried out autocorrelation analysis to characterize SecA-GFP and GFP-SecY dynamics. Autocorrelation function (ACF) carpets revealed a range of diffusion times for SecA-GFP and GFP-SecY and complex behavior with a pronounced presence of delayed peaks.

**Fig 3:**
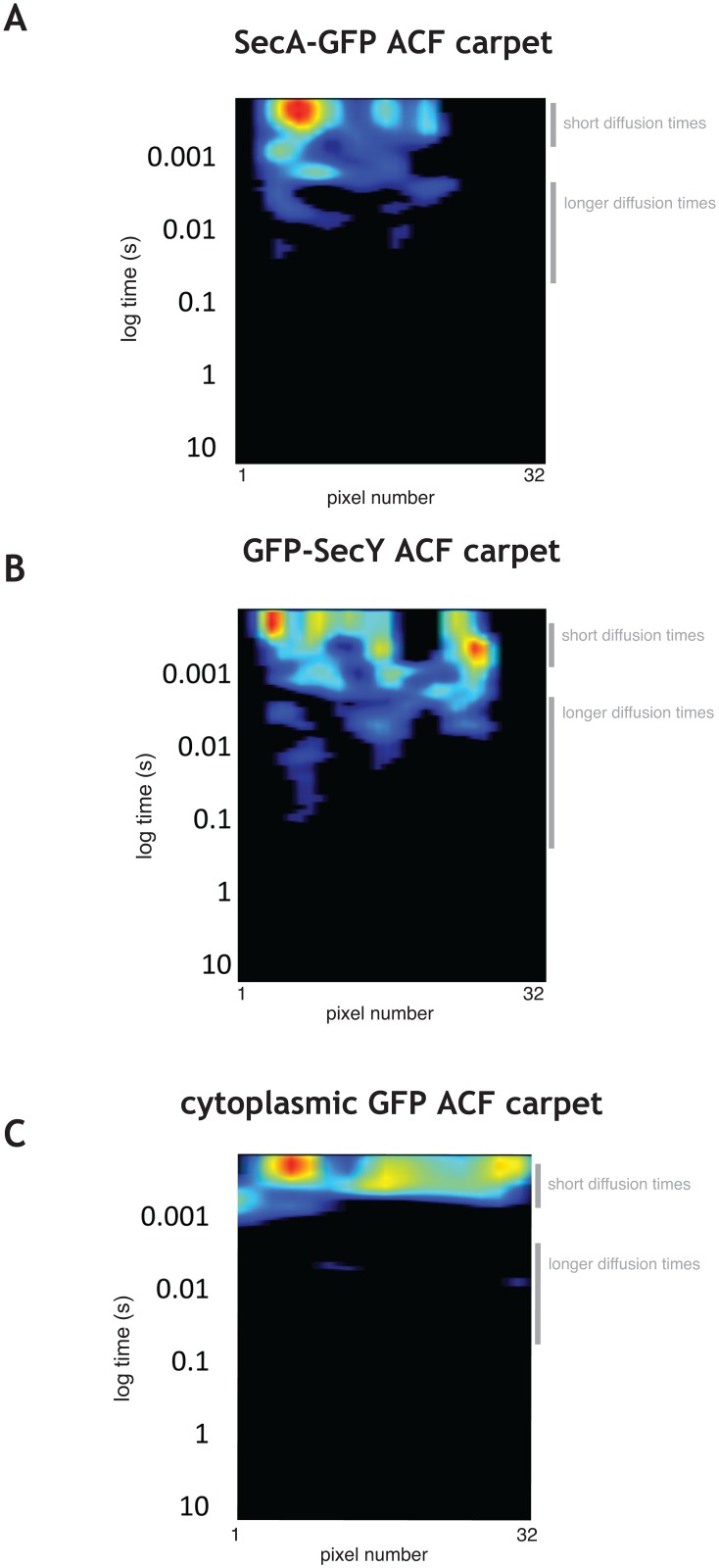
ACF analysis of SecA-GFP, GFP-SecY, and GFP. **A, B,** and **C.** Autocorrelation function (ACF) carpets for a typical exponentially growing *B*. *subtilis* cell expressing SecA-GFP **(A)**, GFP-SecY (strain amyE::pGY1) **(B)**, and cytoplasmic GFP **(C).** For all panels the amplitude of the autocorrelation function in each pixel is shown as a heat map (32 pixels, 80 nm each) plotted against a logarithmic time lag.

Fast diffusing SecA-GFP molecules as well as populations of slower SecA-GFP species (Fig 3A and S6A Fig) were present, the latter suggesting binding of SecA to other proteins (e.g. free SecYEG heterotrimers in the membrane) and/or to larger protein complexes (e.g. active translocases). Similarly, for GFP-SecY there were fast diffusing species (Fig 3B and S6B Fig), consistent with the free membrane population of translocons, i.e. SecY (47 kDa) complexed with its low molecular weight partners SecE (6 kDa) and SecG (7 kDa), or alternatively SecY diffusing alone. The presence of autocorrelation at slower times was more pronounced in the SecY ACF. This behavior is consistent with molecules engaged in repeated interactions with larger complexes, such as in cycles of association-dissociation with translocases during a translocation event. They have longer apparent diffusion times since these cycles of molecular interactions retard their movement (see also Discussion). The two distinct behaviors detected by ACF (free diffusion and repeated binding) are highlighted in S6 Fig, panels B and C. In contrast, the ACF of untagged cytoplasmic GFP exhibited only a restricted range of fast diffusing species (Fig 3C), indicating that the observed behaviors were specific to SecA-GFP and GFP-SecY.

We fitted the autocorrelation curves of SecA-GFP and GFP-SecY to a two component diffusion model to extract diffusion coefficients and the relative abundances of the slow and fast moving species (S7A Fig). The faster diffusing species of SecA-GFP had a diffusion coefficient of 1.2±0.56 μm^2^/s, which is consistent with cytoplasmic diffusion of a SecA-GFP dimer (MW of *B*. *subtilis* SecA = 95 kDa). The bound population had an apparent diffusion coefficient of 0.03±0.03 μm^2^/s, due to the cycles of association-dissociation from larger complexes. The somewhat high variability of the diffusion coefficient of this component is consistent with the diversity of timescales of molecular interactions. The freely diffusing population represented 67% of the total SecA-GFP in the cell, suggesting that the remaining third of cellular SecA is involved in binding interactions with larger assemblies. Interestingly, the relative amplitude of the slow components of GFP-SecY autocorrelation function was more significant than what was observed for SecA-GFP (Fig 3B) suggesting that a larger fraction of SecY than of SecA is involved in binding interactions with larger molecular complexes. Indeed, fitting of the autocorrelation functions to a two species model (S7B Fig) showed that the diffusive and the bound population of SecY each represented about 50±2% of the total. Taken together, our FCS data are consistent with the existence of a large cytoplasmic pool of SecA molecules and a freely diffusive pool of SecY molecules in the membrane, both of which interact dynamically with larger molecular complexes.

### pCF reveals diffusive paths of SecA and SecY in the cell

**Fig 4:**
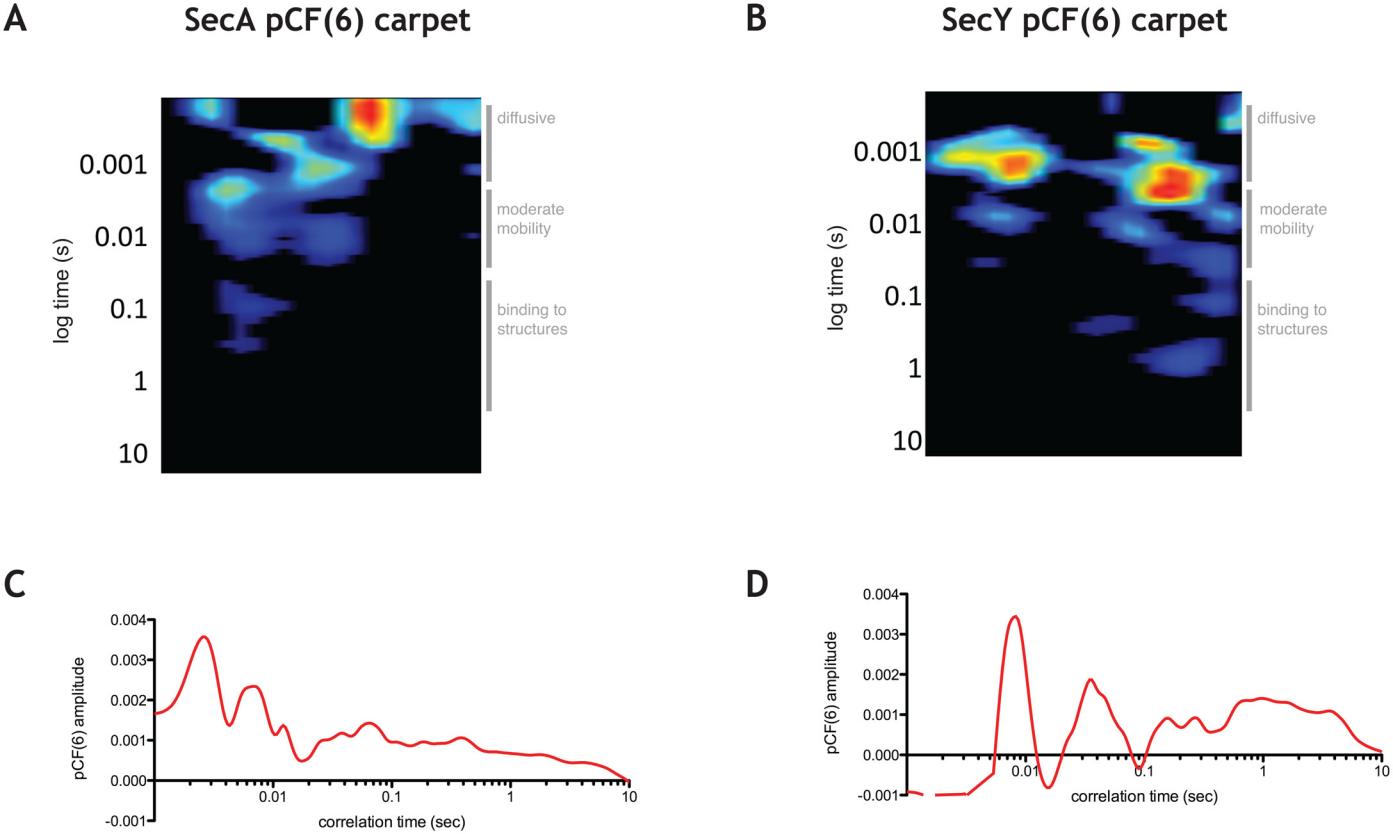
pCF analysis of SecA-GFP and GFP-SecY. **A.** pCF(6) for a typical cell expressing SecA-GFP with the times characteristic for various diffusive species shown on the right of the graph. **B.** pCF(6) for a typical cell expressing GFP-SecY with the times characteristic for various diffusive species shown on the right of the graph. **C.** Average pCF(6) for panel A. **D.** Average pCF(6) for panel B.

In order to characterize the diffusive paths of SecA-GFP and GFP-SecY subpopulations, we undertook pair correlation function (pCF) analysis [23,25,32]. To determine how SecA and SecY molecules traverse the distance between neighboring structures in the cell, we chose to examine pairs of points six pixels apart (pCF(6)) in a given cell, which corresponds to the average distance between SecA-GFP and GFP-SecY foci detected by TIRFM (6 pixels ~ 0.5 μm). pCF(6) of SecA-GFP showed peaks at short times, intermediate times and peaks at longer times (results from a typical cell are shown in Fig 4A and 4C, for more cells see S8A Fig). This suggests that SecA-GFP molecules traverse the average distance of ~0.5 μm in the cell in different manners. The peaks in the short time range correspond to fast diffusing population of SecA-GFP identified by ACF, which is consistent with molecules traversing this distance without interacting with other proteins. The presence of peaks at intermediate times is consistent with a SecA-GFP subpopulation that traverses this distance bound to protein partners and/or while associated with the membrane. Finally, the peaks and the trailing amplitude at very long times are consistent with a subpopulation of molecules of SecA-GFP that traverses this distance while being engaged in association-dissociation with structures immobile on the timescale of the experiment.

Strikingly, the freely diffusing population of GFP-SecY was nearly completely absent ~ 0.5 μm away, as evidenced by the absence of significant amplitudes of pair correlation function at short time lags (a typical cell is shown in Fig 4B and 4D, for more examples see S8B Fig), indicating that the diffusion of free SecY molecules on the membrane detected by ACF is only local. This suggests that SecY and/or SecYEG may become only transiently released from larger structures and protein partners before being recaptured. The peaks of correlation of pCF(6) at intermediate times suggested that SecY traverses this distance while in complex with other proteins, while the peaks at long times indicate its interaction with structures that do not move on this timescale.

For both SecA and SecY, the pCF(2) was qualitatively equivalent to ACF (S8C and S8D Fig), as expected, given that the analysis is done within a single point spread function of the microscope. On the other hand, pCF(14) detected very few molecules either because they didn’t move to this location or more likely because they left the plane of the linescan during acquisition (S8C and S8D Fig).

Taken together, these data suggest that, on average, the transition of SecY molecules between neighboring translocases occurs only when SecYEG is complexed with other proteins, but not alone.

## Discussion

In this work, we have used cutting edge imaging techniques to examine the localization and dynamics of the two core components of the Sec machinery, SecA and SecY, in growing *Bacillus subtilis* cells. Our results reveal a remarkably dynamic secretion system in constant reorganization in the cell. Both SecA and SecY form clusters throughout the cytoplasmic membrane with an average linear spacing of 0.55 micrometers. Such clusters are consistent with the previously reported spatial organization of SecA, SecY and the secretory protein pre-AmyQ in discrete sites in the membrane of *B*. *subtilis* cells, and were proposed to be sites of protein export (i.e. translocases) [14]. In contrast, we found no evidence that translocases are distributed along spiral-like structures running the length of the cell, as previously suggested by Campo and colleagues. On the timescale of one or two minutes both SecA and SecY visit with apparently equal probability the entire surface of the cell, indicating that secretion complexes display no high-order organization and/or directed motion along helical structures in the membrane. This also indicates that translocases are highly dynamic, and thus not attached to large structures, such as the cell wall.

**Fig 5:**
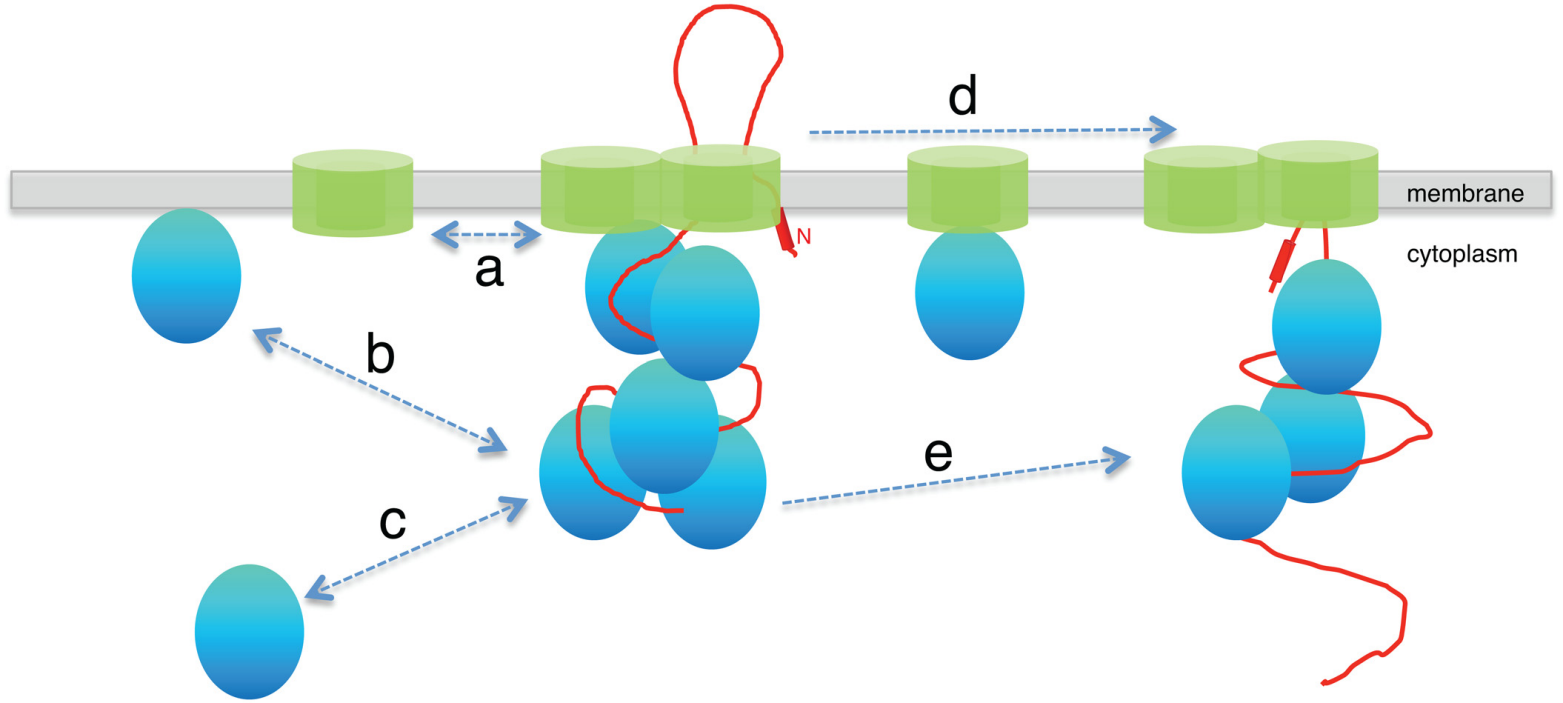
Model of the secretory machinery. The SecYEG translocon (in green) is comprised SecYEG molecules which are engaged in cycles of association and dissociation with the SecY molecules on the membrane (a). SecA (shown in blue) delivers the pre-protein (shown in red) destined for export across the membrane to the translocon, where multiple molecules of SecA associate and accumulate during translocation. This association is dynamic because molecules of SecA exchange with the surrounding free molecules on the membrane (b) and in the cytoplasm (c). The SecYEG heterotrimer moves between neighboring translocons only when complexed with other proteins, such as SecA (d). A single SecA molecule can sequentially engage with neighboring SecYEG complexes (e).

Secretion of polypeptides is believed to occur in the timescale of several seconds [27]. While membrane-associated GFP freely diffused and displayed a homogeneous localization in the cell over 3 s, SecA and SecY still localized in discrete clusters, further supporting the idea that these may correspond to translocases. Interestingly, a diffuse fluorescence signal was specifically detected around the clusters in this timescale (3 s maximum projections). Species of SecA and SecY with diffusion coefficients consistent with freely diffusing proteins were however detected in our scanning FCS analysis. If they were indeed freely diffusing throughout the membrane, these species would display a homogeneous fluorescence signal over the entire TIRF section over 3 s, like membrane-associated GFP does. The concentration of SecA and SecY molecules around the clusters suggests that both membrane-associated SecA and SecY repeatedly and transiently interact with the translocase complexes (‘a’ and ‘b’ in Fig 5). This is consistent with the sFCS analysis which shows molecular species of SecA and SecY whose diffusion times are compatible with binding to protein partners as well as to very slowly moving or largely immobile structures (on the timescale of the experiment).

The pair correlation function (pCF) approach complements sFCS by quantifying average times that molecular species take to traverse a specified distance in the cell. We chose the distance of approximately two point spread functions for our pCF analysis, which corresponds to the average distance between the membrane foci of SecA and SecY (~0.55 μm). Our pCF analysis shows that SecA traverses this distance (i) as a freely diffusing species, (ii) complexed with protein partners, most likely SecY (arrow d in Fig 5), and (iii) while being engaged in binding interactions with larger structures. These structures are consistent with the foci, presumably translocases, that do not diffuse away on this timescale in our TIRF analysis. Because the pCF analysis detects individual molecules, this indicates that the same molecule of SecA interacts with multiple translocases in the cell. A recent *in vitro* study showed that there was a flux of SecA molecules on translocases [11], but couldn’t address the question of whether the same molecule of SecA engages with a given translocase multiple times or whether it interacts with multiple translocons. Our pCF analysis and TIRF imaging provide evidence that, *in vivo*, this flux of SecA molecules involves not only the participation of multiple SecA molecules in a single translocase, but that after dissociating from one translocase SecA molecules seem to engage with neighboring translocases without spending much time freely diffusing.

Surprisingly, pCF analysis of GFP-SecY indicated that freely diffusive SecY single molecules on average do not traverse the distance between two SecY clusters (0.5 μm), indicating that they only have a local existence on the membrane. Thus, free SecY is a transient species that quickly complexes with other protein partners on the cytoplasmic membrane. Because the K_d_ of SecA—SecY interaction is in the nanomolar range (~ 4 nM) [2] and the concentration of SecA in the cell in the micromolar range [33], it is likely that SecY subunits become rapidly complexed with SecA molecules. Assuming a diffusion-limited on-rate for such an interaction of ~ 2*10^6^ M^-1^s^-1^ [34], the off rate would be expected to be only k_off_ ~ 6*10^−3^ s^-1^, indicating a very slowly dissociating complex. Consistently, high molecular weight species with diffusion times consistent with SecYEG-SecA complexes were detected by pCF 0.5 μm apart for both SecA and SecY (‘d’ in Fig 5).

To conclude, this work draws a very dynamic picture of secretion complexes on various timescales in live *B*. *subtilis* cells. While the mechanistic properties of the Sec system have been largely studied *in vitro*, we provide insight of the Sec system in live cells for the first time. Our work shows that TIRF microscopy coupled with methods based on fluorescence autocorrelation spectroscopy are powerful tools to address cell biological questions related to membrane-associated processes not only in eukaryotic systems but also in bacterial cells. It is now possible to use these methods to study association and dissociation constants of proteins *in vivo*, to obtain a quantitative picture of cellular processes that has so far only been *possible in vitro*.

## Experimental Procedures

### Strains and growth conditions

Bacillus subtilis strains used in this study are listed in Table A in S1 File. Cells were grown in Luria-Bertani (LB) medium supplemented with the appropriate antibiotics, when required, at the following concentrations: chloramphenicol, 5 μg/mL; spectinomycin, 100 μg/mL. Cells from frozen stocks were subcultured in small volumes of medium and grown over night. The following day the cells were diluted 5000 fold and grown for several hours until they attained exponential phase. Cells for microscopy were taken at OD_600_ ~ 0.3–0.4. 0.05% xylose was used for induction of GFP-SecY.

### Cloning and strain construction

We used standard procedures for restriction digestion, ligation, agarose gel electrophoresis, and transformation of competent E. coli cells. Chromosomal DNA of *B*. *subtilis* was isolated with the Wizard Genomic DNA Purification Kit (Promega), using the manufacturers instructions. Plasmid DNA from E. coli was isolated using the QIAprep Spin Miniprep Kit (Qiagen), using the manufacturers instructions. Phusion (NEB) DNA polymerase was used for cloning and ExTaq DNA polymerase (Takara) for screening for correct constructs.

In order to create an inducible GFP fusion of the membrane-binding tail of MinD, the coding sequence of the C-terminal fragment of *minD* was PCR-amplified from *B*. *subtilis* 168 chromosomal DNA using primers minD22fwd (CTGTTCTCGAGCAGGTGCTTGAAGAGCAAAACAAAGGAATG), minD22rev (CTATCAAGCTTAGATCTTACTCCGAAAAATGACTTAATCTTAGCC), carrying the XhoI and HindIII restriction sites. The resulting PCR products were purified, digested and ligated to XhoI–HindIII cleaved pSG1154 vector. This resulted in the plasmid pMIND22. The plasmids were isolated and used to transform *B*. *subtilis* 168 and clones were selected on LB agar plates containing spectinomycin. The resulting strains were tested on plates with 1% starch to confirm integration at the amyE locus, restreaked to purify and stored at -80°C.

### TIRF microscopy and image processing

For microscopic observations, 2–3 μL of cells from exponentially growing cultures (OD ~0.3–0.4) were placed on a thin pad of 1% agarose poured in frames glued on microscope slides (Gene Frame from Thermo Scientific) and imaged by an inverted Nikon microscope (Ti-E) with a diode pumped solid-state laser (Cobold Calypso, 50 mW, 491 nm) and an Apo TIRF 100x oil objective (Nikon) with a numerical aperture of NA = 1.49. Images and time-lapse movies were collected with an electron-multiplying charge-coupled device (EMCCD) camera (iXon3 DU-897, Andor) with a gain set at 300. The microscope and the laser were controlled by the Nikon NIS-Elements software. Incidence angles of the laser beam were adjusted to obtain either TIRF or epifluorescence illumination, as indicated in the text.

All images and time-lapse movies were analyzed using ImageJ. To obtain the cell outlines shown in the figures, maximum projection images were produced from time-lapse movies whereupon the maximum projections were thresholded to obtain the cell outlines. To measure the total fluorescence intensities of the membranes visible in the TIRF field, a region of interest (ROI) was created from a cell outline. Fluorescence intensities were thereupon measured for each frame of a movie in this ROI. Fluorescence intensities reported in the figures are normalized to 100. To measure the fluorescence intensities of the foci, we used the function ‘find maxima’ of ImageJ (output type: point selection) to identify the foci. We then measured the fluorescence intensities associated with the foci and calculated the reported distributions. All images shown in the figures were processed as follows: background was subtracted with a rolling ball radius of 50 pixels, the images were filtered using a Gaussian filter with a sigma of 1 pixel. All images are presented with ‘physics’ lookup table, done in ImageJ.

### Acquisition of line-scan data

The microscopy measurements were performed on a Zeiss LSM710 Quasar laser scanning microscope, using a 40X water immersion objective 1.2 N.A. (Zeiss, Germany). GFP was excited with the 488 nm emission line of an Argon laser. GFP was measured using the 510–560 nm emission range and the pinhole was set to 1 Airy Unit. A detailed description of the experimental settings used for the line-scan measurement is present in previous publications [23,25]. Briefly, we acquire data by rapidly scanning a diffraction-limited laser beam (488 nm) along a 32 pixel long line drawn across the long axis of a cell (as shown diagrammatically in figures) expressing SecA-GFP or SecY-GFP. The zoom was set so that 1 pixel = 80 nm. The scanning speed used for these settings was selected (pixel dwell time 10.18 μs, line time 0.763 ms) so that the GFP molecules could be correlated in time between lines. In general for each experiment, 2x10^5^ consecutive lines (with no intervals between lines) were acquired. Time regions within each experiment with no average change in fluorescence intensity (e.g. photo-bleaching) were then selected for the correlation analysis.

### Autocorrelation and pair correlation analysis of line-scan data

Calculation of the auto and pair correlation functions as well as the waist of the point spread function was done using the SimFCS software developed at the Laboratory for Fluorescence Dynamics (www.lfd.uci.edu), as previously described [23,25]. Intensity data are presented by using a carpet representation in which the x-coordinate corresponds to the point along the line (pixels) and the y-coordinate corresponds to the time of acquisition. The autocorrelation function (ACF) and the pair correlation functions (pCF(pixels)) are displayed in pseudo colors in an image in which the x-coordinate corresponds to the point along the line and the y-coordinate corresponds to the autocorrelation time in a log scale. The pCF analysis was carried out at a distance of 5–6 pixels (which corresponds to 500–600 nm).

## Supporting Information

S1 Fig Distribution of SecA-GFP foci in three successive 3 second partial maximum projections after treatment with sodium azide. Maximum projections of successive 3 s segments of a time-lapse TIRF acquisition taken in streaming mode with 100 ms integration time; cell outlines are represented in the top panel. A typical cell is shown with ‘physics’ LUT from FIJI (see Materials and Methods).
(EPS)

S2 Fig Distribution of SecA-GFP foci in successive 3 second partial maximum projections. **A.** Maximum projections of successive 3 s segments of a time-lapse TIRF acquisition taken in streaming mode with 100 ms integration time (a-h); cell outlines are represented in the top panel. A typical cell is shown with ‘physics’ LUT from FIJI (see Materials and Methods). The intensity scale for fluorescence is shown on the right of the cell outline panel; it also serves as a scale bar of 1 μm. **B.** Fluorescence intensity profiles along the line drawn over the long axis of the cell shown for frames *a* and *h*, as shown in blue and red respectively in panel A.
(EPS)

S3 Fig Dynamics of membrane associated GFP. **A.** Frames from a time-lapse TIRF acquisition of MinDtail-GFP expressed from strain RCL237 (*amyE*::*spc Pxyl-gfp-minDtail*) taken in streaming mode with the integration time of 100 ms; top panel represents the cell outline (see Materials and Methods). A typical cell is shown with ‘physics’ LUT from FIJI (see Materials and Methods). Intensity scale for fluorescence is 1 μm. **B.** Maximum projection of a 1 min time-lapse TIRF acquisition of MinDtail-GFP. **C.** Fluorescence intensity profiles over the long axis of cell for frames displayed in panel A. **D.** Integrated fluorescence intensities for the entire surface of the cell visible in the TIRF field from panel B.
(EPS)

S4 Fig Dynamics of SecA association/dissociation from membrane hotspots. **A.** Typical maximum projection of SecA-GFP (*B*. *subtilis* strain secA::pSAG2) from a 3 s segment of a 25 s time-lapse TIRF acquisition. **B.** Evolution of fluorescence intensity as a function of time for the region of interest (ROI) around the hotspot shown in panel A by the white square. **C.** Representative frames were extracted every 0.3 s from the time series to visualize how accumulation/dissipation of SecA-GFP in the hotspot occurs over the timescale of seconds. Images are displayed with ‘physics’ LUT from FIJI (see Materials and Methods).
(EPS)

S5 Fig Membrane localization of GFP-SecY in epifluorescence mode and the distribution of foci in successive 3 second TIRF partial maximum projections. **A.** Epifluorescence image of a mid-section of a cell expressing GFP-SecY (same experimental conditions as in Figs 2, 3 and 4). **B.** Quantification of fluorescence intensity along the green line shown in panel A. **C.** Maximum projections of successive 3 s segments of a 30 s time-lapse TIRF acquisition of exponentially growing cells expressing GFP-SecY, taken in streaming mode with integration time of 100 ms. The top panel are cell outlines (see Materials and Methods). A typical cell is shown with ‘physics’ LUT from FIJI (see Materials and Methods). The intensity scale for fluorescence is shown on the right of the cell outline panel; it serves also as a scale bar of 1 μm. **D.** Fluorescence intensity profiles along the line drawn over the long axis of the cell shown for frames *a* and *j*, as shown in blue and red respectively in panel C.
(EPS)

S6 Fig ACF analysis of SecA-GFP and GFP-SecY. **A and B.** ACF carpets of three exponentially growing *B*. *subtilis* cell expressing SecA-GFP **(A)** and GFP-SecY **(B)**; the amplitudes of the autocorrelation functions in each pixel are shown as a heat map. Experimental conditions identical to those in Figs 2, 3 and 4. **C.** Autocorrelation functions for the pixels indicated by the yellow and red arrows in panel B. The typical diffusion times for free diffusion and the bound subpopulation of molecules are indicated.
(EPS)

S7 Fig ACF fits for cells expressing SecA-GFP and GFP-SecY. **A and B.** Autocorrelation function plot averaged for six cells expressing SecA-GFP (**A**) and GFP-SecY (**B**) with the fitted curve shown in green. Experimental conditions were as in Fig 3.
(EPS)

S8 Fig pCF analysis of SecA-GFP and GFP-SecY. **A and B.** pCF(6) analysis for three exponentially growing *B*. *subtilis* cells expressing SecA-GFP (**A**) and GFP-SecY (**B**). Experimental conditions were as in Fig 3. **C.** and **D.** Comparison of ACF, pCF(2), pCF(6), and pCF(14) for SecA (C), and SecY (D).
(EPS)

S1 File Supporting Information. Supplementary tables, results and references.
(DOC)

S1 Video Movie showing dynamics of SecA-GFP. TIRFM acquisition with 100 ms integration time. The AVI file was created with 10 FPS setting.
(AVI)

S2 Video Movie showing dynamics of GFP-SecY. TIRFM acquisition with 100 ms integration time. The AVI file was created with 10 FPS setting.
(AVI)
