# Supplementary material for: Dynamic Organization of SecA and SecY Secretion Complexes in the B. subtilis Membrane
Source: PLoS One. 2016 Jun 23;11(6):e0157899. doi: 10.1371/journal.pone.0157899 (PMC4918944; doi:10.1371/journal.pone.0157899)
Supplement: S1 File — Supplementary tables, results and references. (DOC) [file pone.0157899.s009.doc]

**Supporting Information**

**Cytosolic SecA molecules dynamically interact with membrane-associated SecA foci**

To further support the hypothesis that the cytoplasmic SecA molecules dynamically interacted with SecA clusters in the membrane, we measured the coefficient of variation of fluorescence intensity of the entire cell surface visible in TIRFM over a longer period (30 s). A higher coefficient of variation indicates significant deviations from the mean fluorescence intensity over time, as expected if SecA-GFP molecules were entering and exiting the TIRF field due to interaction with membrane protein complexes. The coefficient of variation of fluorescence intensity was significantly greater in cells expressing SecA-GFP than in cells expressing MinD_tail_-GFP or GFP-SecY (Table B). Furthermore, the coefficient of variation of SecA-GFP was greatly diminished when the translocation ATPase function of SecA was specifically inhibited by sodium azide **[1]**, or when the level of membrane-associated SecA was decreased by dissipation of the pmf **[2]** using the ionophore carbonyl cyanide m-chlorophenylhydrazone (CCCP) (Table B). This indicates that the coefficient of variation was measuring fluorescence fluctuations specific to SecA-GFP.

**MinDtail-GFP molecules stably associate with the membrane**

As a control for the detection of reversible interaction of SecA-GFP with the membrane using TIRFM, we looked at the total fluorescence intensity of GFP stably associated with the membrane through the membrane-binding tail of Bacillus subtilis MinD (MinDtail), which is composed of an amphipathic helix sufficient to attach proteins to the membrane [3]. This construct would not be expected to enter and exit the TIRF field and would not therefore show fluorescence fluctuations similar to SecA-GFP. GFP-MinDtail did not form foci in the membrane and was homogeneously distributed on all timescales (S2 Fig). This indicates that the organization of SecA-GFP in membrane foci is not a generic property of membrane-localized proteins in *B. subtilis*, as may be the case in yeast where most membrane proteins organize in distinct membrane domains [4]. As expected, although there was a slight decrease in the fluorescence signal due to photobleaching, the total fluorescence intensity of the membrane surface of cells expressing this construct did not show the fluorescence fluctuations we observed with SecA-GFP over the same number of frames of a 700 ms time-lapse movie, (S2 Fig and Fig 1F). This was in contrast to the fluorescence signal from cells expressing SecA-GFP, which fluctuated significantly over the same number of frames (Fig 1F).

# Supplementary Figure Legends

**S1 Fig. Distribution of SecA-GFP foci in three successive 3 second partial maximum projections after treatment with sodium azide.** Maximum projections of successive 3 s segments of a time-lapse TIRF acquisition taken in streaming mode with 100 ms integration time; cell outlines are represented in the top panel. A typical cell is shown with ‘physics’ LUT from FIJI (see Materials and Methods).

**S2 Fig. Distribution of SecA-GFP foci in successive 3 second partial maximum projections. A.** Maximum projections of successive 3 s segments of a time-lapse TIRF acquisition taken in streaming mode with 100 ms integration time (a-h); cell outlines are represented in the top panel. A typical cell is shown with ‘physics’ LUT from FIJI (see Materials and Methods). The intensity scale for fluorescence is shown on the right of the cell outline panel; it also serves as a scale bar of 1 μm. **B.** Fluorescence intensity profiles along the line drawn over the long axis of the cell shown for frames *a* and *h*, as shown in blue and red respectively in panel A.

**S3 Fig.** **Dynamics of membrane associated GFP. A.** Frames from a time-lapse TIRF acquisition of MinDtail-GFP expressed from strain RCL237 (*amyE::spc Pxyl-gfp-minDtail*) taken in streaming mode with the integration time of 100 ms; top panel represents the cell outline (see Materials and Methods). A typical cell is shown with ‘physics’ LUT from FIJI (see Materials and Methods). Intensity scale for fluorescence is 1 μm. **B.** Maximum projection of a 1 min time-lapse TIRF acquisition of MinDtail**-**GFP. **C.** Fluorescence intensity profiles over the long axis of cell for frames displayed in panel A. **D.** Integrated fluorescence intensities for the entire surface of the cell visible in the TIRF field from panel B.

**S4 Fig. Dynamics of SecA association/dissociation from membrane hotspots. A.** Typical maximum projection of SecA-GFP (*B. subtilis* strain secA::pSAG2) from a 3 s segment of a 25 s time-lapse TIRF acquisition. **B.** Evolution of fluorescence intensity as a function of time for the region of interest (ROI) around the hotspot shown in panel A by the white square. **C.** Representative frames were extracted every 0.3 s from the time series to visualize how accumulation/dissipation of SecA-GFP in the hotspot occurs over the timescale of seconds. Images are displayed with ‘physics’ LUT from FIJI (see Materials and Methods).

**S5 Fig. Membrane localization of GFP-SecY in epifluorescence mode and the distribution of foci in successive 3 second TIRF partial maximum projections. A.** Epifluorescence image of a mid-section of a cell expressing GFP-SecY (same experimental conditions as in Figures 2, 3, 4). **B.** Quantification of fluorescence intensity along the green line shown in panel A. **C.** Maximum projections of successive 3 s segments of a 30 s time-lapse TIRF acquisition of exponentially growing cells expressing GFP-SecY, taken in streaming mode with integration time of 100 ms. The top panel are cell outlines (see Materials and Methods). A typical cell is shown with ‘physics’ LUT from FIJI (see Materials and Methods). The intensity scale for fluorescence is shown on the right of the cell outline panel; it serves also as a scale bar of 1 μm. **D.** Fluorescence intensity profiles along the line drawn over the long axis of the cell shown for frames *a* and *j*, as shown in blue and red respectively in panel C.

**S6 Fig. ACF analysis of SecA-GFP and GFP-SecY. A and B.** ACF carpets of three exponentially growing *B. subtilis* cell expressing SecA-GFP **(A)** and GFP-SecY **(B)**; the amplitudes of the autocorrelation functions in each pixel are shown as a heat map. Experimental conditions identical to those in Figures 2, 3, and 4. **C.** Autocorrelation functions for the pixels indicated by the yellow and red arrows in panel B. The typical diffusion times for free diffusion and the bound subpopulation of molecules are indicated.

**S7 Fig. ACF fits for cells expressing SecA-GFP and GFP-SecY. A and B.** Autocorrelation function plot averaged for six cells expressing SecA-GFP (**A**) and GFP-SecY (**B**) with the fitted curve shown in green. Experimental conditions were as in Figure 3.

**S8 Fig. pCF analysis of SecA-GFP and GFP-SecY. A and B.** pCF(6) analysis for three exponentially growing *B. subtilis* cells expressing SecA-GFP (**A**) and GFP-SecY (**B**). Experimental conditions were as in Figure 3. **C.** and **D.** Comparison of ACF, pCF(2), pCF(6), and pCF(14) for SecA (C), and SecY (D).

**S1 Video. Movie showing dynamics of SecA-GFP.** TIRFM acquisition with 100 ms integration time. The AVI file was created with 10 FPS setting.

**S2 Video. Movie showing dynamics of GFP-SecY.** TIRFM acquisition with 100 ms integration time. The AVI file was created with 10 FPS setting.

**Table A. Table of strains used in this study**

| **strain** | **genotype** | **reference** |
| --- | --- | --- |
|  |  |  |
| secA::pSAG2 | *trpC2 Ω(secA::secA-gfp cat)* | Campo et al. (2004) [6] |
| amyE::pGY | *trpC2 Ω(amyE::pGY1(spc pxyl-gfp-secY)* | Campo et al. (2004) |
| RCL237 | *trpC2 Ω(amyE::spc pxyl-gfp-minDtail)* | this work |
| ASR111 | *trpC2 Ω(amyE::pP_xyl_ gfp spc* | Rueff et al (2014) [5] |

**Table B. Coefficient of variation of integrated fluorescence intensities of the entire cell membrane for 30 second segments of time-lapse TIRF acquisitions**

|  | **MinDtail-GFP** | **SecA-GFP** | **GFP-SecY** | **SecA-GFP +azide** | **SecA-GFP +CCCP** |
| --- | --- | --- | --- | --- | --- |
| coefficient of variation (integrated fluorescence intensity) | 8.5±1%  (n=23) | 24±3% (n=25) | 11±3%  (n=25) | 16±2%  (n=28) | 16±2%  (n=25) |

**REFERENCES**

1. Oliver DB, Cabelli RJ, Dolan KM, Jarosik GP (1990) Azide-resistant mutants of Escherichia coli alter the SecA protein, an azide-sensitive component of the protein export machinery. Proc Natl Acad Sci U S A 87: 8227-8231.

2. Nishiyama K, Fukuda A, Morita K, Tokuda H (1999) Membrane deinsertion of SecA underlying proton motive force-dependent stimulation of protein translocation. EMBO J 18: 1049-1058.

3. Szeto TH, Rowland SL, Habrukowich CL, King GF (2003) The MinD membrane targeting sequence is a transplantable lipid-binding helix. J Biol Chem 278: 40050-40056.

4. Spira F, Dominguez-Escobar J, Muller N, Wedlich-Soldner R (2012) Visualization of cortex organization and dynamics in microorganisms, using total internal reflection fluorescence microscopy. J Vis Exp: e3982.

5. Rueff AS, Chastanet A, Dominguez-Escobar J, Yao Z, Yates J, et al. (2014) An early cytoplasmic step of peptidoglycan synthesis is associated to MreB in Bacillus subtilis. Mol Microbiol 91: 348-362.

6. Campo N, Tjalsma H, Buist G, Stepniak D, Meijer M, et al. (2004) Subcellular sites for bacterial protein export. Mol Microbiol 53: 1583-1599.
